# Textile Masks and Surface Covers—A Spray Simulation Method and a “Universal Droplet Reduction Model” Against Respiratory Pandemics

**DOI:** 10.3389/fmed.2020.00260

**Published:** 2020-05-27

**Authors:** Alex Rodriguez-Palacios, Fabio Cominelli, Abigail R. Basson, Theresa T. Pizarro, Sanja Ilic

**Affiliations:** ^1^Division of Gastroenterology and Liver Diseases, Case Western Reserve University School of Medicine, Cleveland, OH, United States; ^2^Digestive Health Institute, University Hospitals Cleveland Medical Center, Cleveland, OH, United States; ^3^Department of Pathology, School of Medicine, Case Western Reserve University, Cleveland, OH, United States; ^4^Human Nutrition, Department of Human Sciences, College of Education and Human Ecology, The Ohio State University, Columbus, OH, United States

**Keywords:** coronavirus, respiratory pandemic, COVID-19, SARS-Cov-2, cloth masks, textiles, public droplet safety, spray simulation model

## Abstract

The main form of COVID-19 transmission is via “oral-respiratory droplet contamination” (droplet: very small drop of liquid) produced when individuals talk, sneeze, or cough. In hospitals, health-care workers wear facemasks as a minimum medical “*droplet precaution*” to protect themselves. Due to the shortage of masks during the pandemic, priority is given to hospitals for their distribution. As a result, the availability/use of medical masks is discouraged for the public. However, for asymptomatic individuals, not wearing masks in public could easily cause the spread of COVID-19. The prevention of “environmental droplet contamination” (EnvDC) from coughing/sneezing/speech is fundamental to reducing transmission. As an immediate solution to promote “**public droplet safety**,” we assessed household textiles to quantify their potential as effective environmental droplet barriers (EDBs). The synchronized implementation of a universal “community droplet reduction solution” is discussed as a model against COVID-19. Using a bacterial-suspension spray simulation model of droplet ejection (mimicking a sneeze), we quantified the extent by which widely available clothing fabrics reduce the dispersion of droplets onto surfaces within 1.8 m, the minimum distance recommended for COVID-19 “social distancing.” All textiles reduced the number of droplets reaching surfaces, restricting their dispersion to <30 cm, when used as single layers. When used as double-layers, textiles were as effective as medical mask/surgical-cloth materials, reducing droplet dispersion to <10 cm, and the area of circumferential contamination to ~0.3%. The synchronized implementation of EDBs as a “community droplet reduction solution” (i.e., face covers/scarfs/masks and surface covers) will reduce COVID-19 EnvDC and thus the risk of transmitting/acquiring COVID-19.

## Introduction

The main form of COVID-19 transmission is via “*oral-respiratory droplets”* produced when individuals talk, sneeze, or cough. Despite the magnitude of the COVID-19 pandemic, it is disconcerting that the general public either does not have personal protective equipment available to them, including respiratory masks, or chooses to not use them, to contain the pandemic. Worldwide, health-care workers wear medical masks as a minimum “*droplet precaution*” to protect themselves. However, experts appealed to the community not to wear medical masks stating they are not effective for the public (1); albeit, counter-criticisms ensued (2). Regardless of clinical presentation, COVID-19 transmits person-to-person, including children (3), via “oral-respiratory droplets” produced when individuals talk or sneeze/cough. Aside from Asia (4), there are no global guidelines promoting wearing masks in public to control respiratory pandemics (5–10), and no scientific data/guidelines exist promoting masks as a “droplet precaution” for the public (5, 9, 11).

COVID-19 is caused by a novel coronavirus strain (SARS-CoV-2), for which there is no treatment (12, 13). Disease is characterized by fever, coughing/sneezing, dyspnea, and pneumonia, and can lead to death in some cases (14); however, important for asymptomatic transmission, cases increasingly present with gastrointestinal symptoms, and/or fatigue, without fever (15). Regardless of the clinical presentation, COVID-19 transmits person-to-person through oral-respiratory droplets produced when infected individuals [symptomatic or asymptomatic, including children (3)] talk/cough/sneeze, contaminating the environment.

Although viruses can become airborne dust/aerosols, as micro-droplets evaporate, viruses rapidly loose infectivity in the air (half-life = 1 h) (16–20). By contrast, virus survival increases when liquid droplets contaminate surfaces, especially plastic and stainless steel, with long half-lives of 7 and 6 h, respectively (cardboard, 4 h; copper, 1 h) (16). Since COVID-19 transmits when droplets reach the nose/mouth/eyes (21), or when people touch their nose/mouth/eyes after touching droplet-contaminated surfaces [supermarkets/elevators (22)], it is critical to implement strategies to prevent/reduce environmental droplet contamination (EnvDC). This is particularly true for plastic or metal surfaces, which remain infective for days. Herein, we investigated whether common household textiles can be used as environmental droplet barriers (EDBs; facemasks/covers/scarfs, or surface covers) to prevent EnvDC, improve public droplet safety, and support the synchronized implementation of an environmentally-purposed *Universal Droplet Reduction Model* within the public to control respiratory pandemics such as COVID-19.

## Methods

### Simulation of Bacteria-Containing Micro-/Macro-Droplet Clouds

Since viruses exist in association with bacteria and host cells within electrolytes-rich respiratory fluids (23, 24), we used a bacterial-suspension strategy to quantify the number of droplets that could not be visualized, but that could escape textile barriers and cause long-/short-range surface contamination. To enumerate bacteria-carrying micro-droplets, we used household spray bottles filled with an aqueous suspension of 12-probiotic-cultured dairy product (*Lactobacillus lactis, L. rhamnosus, L. plantarum, L. casei, L. acidophilus, Leuconostoc cremoris, Bifidobacterium longum, B. breve, B. lactis, Streptococcus diacetylactis*, and *Saccharomyces florentinus*, 75 ml; 3 × 10^6−7^ cfu/ml, 25 ml Saliva 10^6−7^) in 1,000 ml PBS (Fisher BP-399-1) to simulate a cloud of droplets produced by a sneeze. Probiotics are BSL-1/“Generally Recognized As Safe” by the FDA and all experiments were conducted in BSL-2 HEPA-filtered microbiology laboratories. No animal/human subjects were used for experimentation. Before testing, spray bottle nozzles were adjusted to produce cloud and jet-propelled droplets that match the visual architecture of droplet formation described by Bourouiba (23). Specifically, we used a high-volume trigger single-v-orifice nozzle sprayer (1.0 ml per stroke) with a 28/400 neck and 9-1/4-inch dip tube fitted with a filter screen (model PA-HDTS-EA, Mfr. Model # 922HL, Delta Industries, Inc.). Using infrared imaging we recently illustrated that the spray model was composed of various liquid droplet dynamic phases occurring within a single spray (25), which reproduces results in a wide arrange of droplet sizes (previously described as right skewed distribution ranges between 20 and 900 μm, with peak at 70–100 μm) (26), and therefore distance reach and landing velocities. In context, the size of droplets in the human sneeze ranges between 40 and 900 μm, with most droplets (70–100%) normally or bimodally distributed around 360–390 μm (27). The spray bottle ejects fluid with pressures that can reach sufficient pressure (e.g., 10 psi for garden sprayers) to create a short burst of fluid/jet and fan clouds of microdroplets. In context, the pressure during a sneeze is 1 psi in the trachea, and 2.6 psi in the mouth/pharynx, while exhalation during strenuous activity triggers a tracheal pressure of 0.03 psi (28). In this model, one stroke ejects 1 ml of fluid per spray, therefore three sprays (delivered at 1 stroke/second) constitute an exposure of 3 ml of fluid in 3 s, which is a delivery of moisture 181-fold faster than the rate of moisture released by the lungs during normal breathing (>20 ml/hour, i.e., equivalent to 5.5 μl/second) (29).

Quantification of droplets landing over a surface was performed at the time of spray using seven 10 mm-Petri dishes containing tryptic soy agar (56.75 cm^2^ surface area/dish) with 5% defibrinated sheep blood, placed on a table spaced at 30 cm intervals between 0 and 180 cm. Plates remained open for 10 min to allow droplet landing. Droplet quantification was conducted for each bottle in duplicate. Large-drop quantification outside agar plates was facilitated by a white droplet footprint left on black surfaces. To test the role of surface covers for unanimated surfaces, Petri dishes were covered with textiles.

### Quantification of Droplet Retention by Household Textiles

To simulate the function of mask barriers, we placed selected textiles (~22 × 22 cm) over a cardboard/plastic-covered 25 × 30 cm surface, over a carved (8.5 × 11 cm) window, and 8.5 cm above the agar plates' plane, through which droplets were sprayed. To avoid altering permeability, textiles were not “tensed” across the carved window. The spray nozzle was placed perpendicular at 8.5 cm from the textile [half the distance between the nostrils and vocal cords, 16–18 cm (30), or one-third of the lip-to-carina distance, 21.6–24.3 cm (31) in humans]. On the other side of the textile, 3–5 agar plates were aligned to cover the 0–8.5, 8.5–17, 17–25.5, and 25.5–34 cm intervals to quantify bacteria-containing droplets that could contaminate a surface. Quantification represents droplets that pass through the textile and that land on a rectangular area of 8.5 cm × 180 cm (agar plate diameter X “spray path”). To quantify the effect of textiles retaining vertically-landing droplets, we quantified droplets reaching agar plates covered with a household textile. All testing conditions were carried out at constant ambient conditions.

### Household Textiles Tested, Replication of Findings, Safety and Contextualization

We first tested six household textiles, including 100% combed cotton (widely available, “T-shirt material”), 100% polyester microfiber 300-thread count fabric (pillow case), two loosely woven “homespun” 100% cotton fabrics (140GSM, 60 × 60-thread count; and 115GSM, 52 × 48-thread count), and “dry technology” 100% polyester common in sport jerseys. These textiles were compared to: (i) the lack of a textile barrier (no mask control), (ii) medical masks, and (iii) surgical cloth material as “gold standard” protective controls. To ensure external validity/reproducibility, complementary and repeated experiments were conducted with selected textiles (i.e., respiratory mask, sports jersey, and Cotton-T-shirt) at the Ohio State University. To contextualize the retention ability of textiles of respiratory secretions, a single episode of a simulated cough by one of the volunteer investigators onto three agar plates, placed perpendicularly at 30 cm inside a BSL-2 safety cabinet, was used to illustrate that respiratory secretions have large strings of mucus more amenable for retention than liquid microdroplets, and which contain bacteria (CFU) recoverable in the TSA agar used in the study. To determine the percentage of area covered by the textile that could be freely open to the direct flow of air liquid macro and microdroplets, we used image analysis of transillumination captures and ImageJ software (https://imagej.nih.gov/ij/). In short, single-/double-layer textile RGB JPG images imported to ImageJ were converted to type 8-bit format, then binary with black background, with threshold adjusted to W190:B255. The quantification of the number of white pixels (background transillumination) for the total image area was then used to compute the percent area of textile that freely allowed the passage of light.

### Statistical Analysis

Student's-*T* tests, linear regression, and multinomial logistic regression were conducted using raw and Log_2_ transformed CFU data (STATA, v15.1). Confidence intervals are provided to convey information relevant to sample size and external validity. Note that the studies represent a large number of simulations shown to be statistically significant. To further ensure external validity and comparability, we derived linear polynomial regression equations that almost perfectly fit the raw data dynamics, *R*^2^ > 0.98, to enable others to adjust the spray droplet landing dynamics on surfaces. ImageJ textile data for single-/double-layer textiles were analyzed using paired *t*-test. Quantitative effects and models were deemed significant if adjusted *p* < 0.05.

### Preprint

This manuscript was submitted to medrxiv on March 29, 2020, and posted as a preprint (32) on April 10 to enable the incorporation of community comments into the peer-review process. In support of this report, peer-reviewers provided an average score of 4.5/5 for six items on the initial submission (originality, and significance to the field, 4.7 ± 0.6 each; rigor, 4.3 ± 0.6; interest to the general audience, 5.0 ± 0; quality of writing, 4.0 ± 1.0; and overall quality of study, 4.3 ± 0.6); and no negative criticisms were publicized for the preprint (tweets from 11 independent accounts with 59,855 followers; April 10–22, 2020).

## Results

### Spray Dispersion Model of Droplets Reach >1.8 Meters if Upward

Because viruses replicate intracellularly in bodily fluids, in association with other microorganisms (23, 24), and need droplets to facilitate their expulsion, transmission, and EnvDC (12), we first validated a rapid spray-simulation model of droplets (mimicking a sneeze) using a bacterial-suspension to quantify the extent by which widely-available household textiles reduced the ejection/long-distance flight of droplets. To facilitate the enumeration of macro-droplets and invisible micro-droplets, spray-simulations were conducted over nutritious-media agar surfaces and incubated for 24 h to enable colony-forming-droplet-unit (CFU) formation.

**Figure 1:**
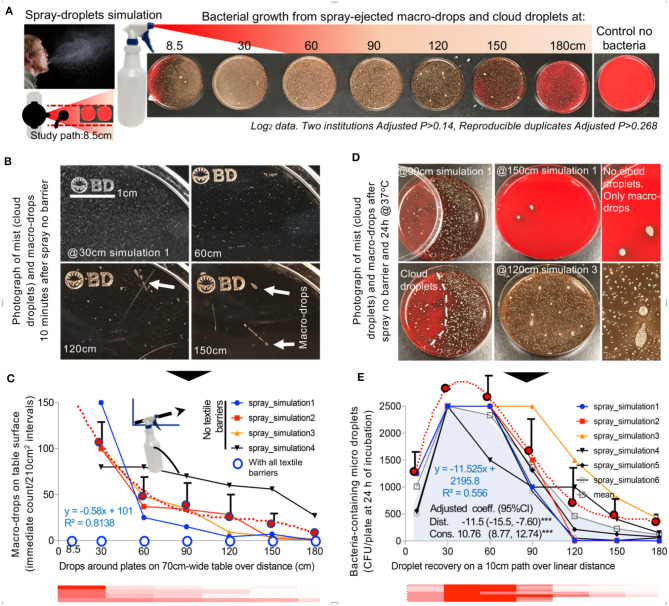
Simulation of a cloud of airborne bacteria-containing macro-drops and micro-droplets to quantify barrier potential of household textiles. **(A)** Graphical overview of the spray model. Inset, Photograph of a human sneeze, public domain, James Gathany, CDC image ID11162). **(B)** Photographs of short and long-range visible droplets immediately after spray. Note the color, number, size, and relative location and distribution of the bacteria colonies growing from “invisible” microdroplets (CFU) shown as whitish spots on the agar surface. Bacterial growth alters the red color of the fresh non-inoculated agar leading to a brownish discoloring of the petri agars, which is more pronounced as the number of bacterial colonies increase. **(C)** Number of macro-drops for four simulations over distance. The overall linear equation that best describes the mean spray macro-droplet dynamics linearized/depicted as the heatmap is *y* = −8E^−05^*x*^3^ + 0.0305*x*^2^ – 3.9405*x* + 198.42, with an *R*^2^ = 0.9829. Note that large drops of liquids observed with the spray alone (no textile barrier) were not observed with any of the textile barriers tested. **(D)** Photographs of bacterial CFUs on agar plates illustrating ability of cloud micro-droplets to move around spaces driven by cloud turbulence (left images, agar plates were partially covered with lid at moment of spray), concurrent contamination with macro- and micro-droplets. **(E)** Number of CFU/plate (56.75 cm^2^) for 6 simulations over distance. The overall linear equation that best describes the mean dispersal of bacteria-carrying micro-droplets over distance, also depicted as the red heatmap, is *y* = −4E^−05^*x*^4^ + 0.0177*x*^3^ – 2.8522*x*^2^ + 155.63*x* – 58.504, with an *R*^2^ = 0.9994.

Based on simulations conducted in two institutions, a cloud of bacteria-carrying droplets travel distances reaching >180 cm, particularly for large droplets (Figure 1A), which is consistent with reported dynamics during sneezing (23). Of relevance to sneezing behavior, simulations illustrate that upward inclination of the central-spray angle allows macro-droplets to reach longer distances (simulation 4/dispersion equations; Figures 1B–E). Although macro-droplets frequently reached 180 cm, most micro-droplets landed on surfaces within 120 cm, with spray air-turbulence carrying micro-droplets into areas not reached with gravity alone. Thus, social distancing of 1.8 m without EDB-mask protection, as is currently recommended, is not always possible and therefore insufficient to prevent droplet exposure, particularly where essential-service workers congregate (i.e., person-person distance is <1.8 m) during pandemics (transportation, supermarkets/food displays). Therein, wearing EDB-masks together with inclining the head/body downward during sneezing could minimize the spatial range of EnvDC.

### Household Textiles Retain Liquid Droplets, Particularly if Double Layered

**Figure 2:**
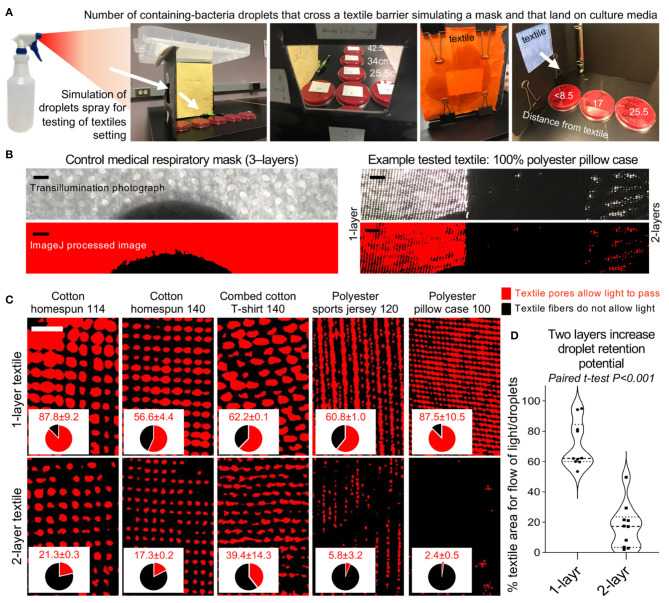
Spray-droplet model to quantify reduction rate of long-range droplet dispersion across 1- and 2-layer textiles. **(A)** Graphical overview of spray-droplet setting (see Methods). Tryptic soy agar supplemented with 5% defibrinated sheep blood plates incubated aerobically at 37°C for 24 h. **(B)** Photograph and low-resolution ImageJ processed image compares medical mask material to that of single- and double-layered textile example (Supplementary Figure 1, all textiles used). Scale bar, 1 mm. **(C)** High resolution ImageJ binary analysis of representative textiles photographed as single and double layers to illustrate the percentage of the textile barrier “open area” that allows the passage of light/droplets. Scale bar, 1 mm. **(D)** Paired analysis of reduction of the textile “open area” when textile is tested as two layers.

**Figure 3:**
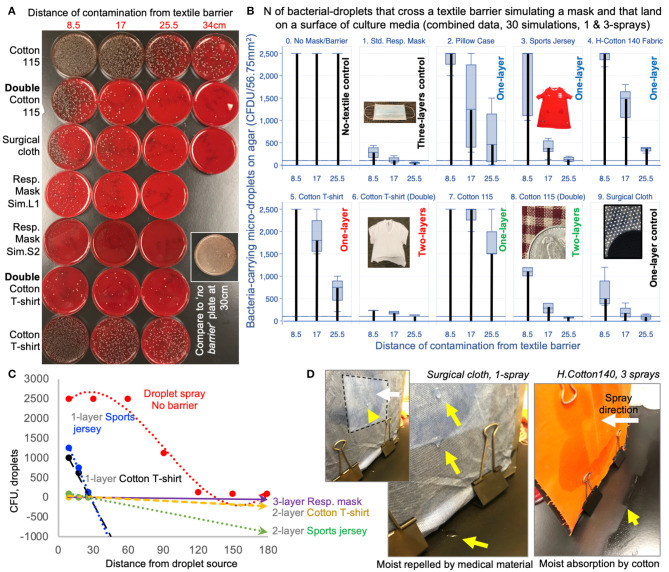
Using two layers of household textiles markedly retain liquid droplets. **(A)** Tryptic soy agar plates illustrate effective bacterial-droplet reduction by 2-layer textiles. **(B)** Pooled results from two spray-simulations (1- and 3-sprays; Supplementary Figure 2). Vertical thick black bars connect baseline values at 0 to the means. **(C)** Linear regressions for EnvDC reduction over distance for no-barrier vs. selected textiles. Compared to no textile (EDB) barrier (red dotted line), the reduction in CFUs illustrate the profound effect of using household textiles to retain droplets. Line slopes that are closer to the horizontal grid line at 0, and closer to the “Resp. mask”-dotted line are more effective strategies (commercial masks are made of 2-or-3-layers) compared to single layers (Supplementary Figure 4, equations and *R*^2^). **(D)** Photographs of differences in condensate after 1-spray on the side of the textile being sprayed. Arrowheads, drops/accumulation.

To quantify the droplet retention potential of textiles as EDBs, we next used the same bacterial-spray-simulation model to quantify non-visualizable micro-droplets that could cross/escape the textile-EDB and cause microbial-surface agar contamination (Figure 2A). Details on textile threading, percentage of area open for flow of droplets/light, and density in grams per square meter (GSM) for all medical and the single-/double-layer household textiles are shown in Figures 2B–D and Supplementary Figure 1. Textiles were tested for one- and three-sprays to determine if EnvDC changed with textile humidity. Although humidity had no statistical impact (dry-vs.-humid, adj.–*P* > 0.2), all textiles, tested as “single-layers,” significantly and reproducibly (between institutions) reduced the ejection of macro-droplets, and the traffic of micro-droplets to <25.5–34 cm (linear regression model adj.–*P* < 0.001, compared to 180 cm with no textile barrier; Figures 3A,B and Supplementary Figures 2, 3).

Remarkably, spray experiments with “two-layers” (of 100%-combed cotton, common in t-shirts; and 100% polyester, in sports jerseys) completely prevented the ejection of large macro-droplets (100% EnvDC prevention), and drastically reduced the ejection of micro-droplets by a factor of 5.16Log_2_, which is equivalent to a 97.2% droplet reduction (*P* < 0.020 vs. single-layers, Figure 3C and Supplementary Figures 4, 5). Importantly, the least-effective textile as single-layer (most-“breathable,” 100%-cotton homespun-115 material) achieved a 90–99.998% droplet retention improvement when used as two-layers (95% CI = 3.74–15.39 Log_2_). Lastly, all textiles were equally effective at absorbing the humidity from 3-sprays compared to medical mask/surgical cloth materials, which condensate after 1-spray (Figure 3D). Together, experiments indicate that two-layers of household textiles are as effective as medical masks preventing EnvDC, and that more breathable materials in ≥2-layers could be effectively used if individuals deem two-layer, “denser” textiles too air-restrictive.

### “Universal Droplet Reduction Model” Against Rapid Respiratory Pandemics

We then rationalized the potential impact of a “universal droplet reduction model” against pandemics, where the community act together to reduce the spatial range of EnvDC. Since it is unclear how many viral particles in droplets (virus/μm^3^) or surfaces (virus/cm^2^) are needed to acquire COVID-19, we assumed that any droplet on a surface area of 56.75 cm^2^ (an 8.5 cm diameter agar plate) renders a surface infective. Since textiles prevented droplets from reaching beyond a ~30 cm radius, we propose a working “droplet reduction model” to control COVID-19, where EDB-masks could reduce the “circumferential area of contamination” around each individual by 97.2% when used as single-layers, or as much as 99.7% when used as two-layers. 100%-cotton/polyester especially shortened the EnvDC radius to <10 cm (similar to medical-mask material; Log_2_ difference = 0.06, for 100% polyester, multinomial adj.–*P* > 0.6). Because COVID-19 cases increase daily, and the fabrication of EDB by centralized organizations could take weeks to reach entire “lockdown” communities, we suggest, based on the cotton/polyester EnvDC effectiveness, and a homemade EDB-mask fabrication trial (Supplementary Figure 6), that, from one piece of clothing, every individual could make (without a sewing machine) two 2-layer-EDB masks as an immediate, synchronized contribution to reduce COVID-19 EnvDC.

**Figure 4:**
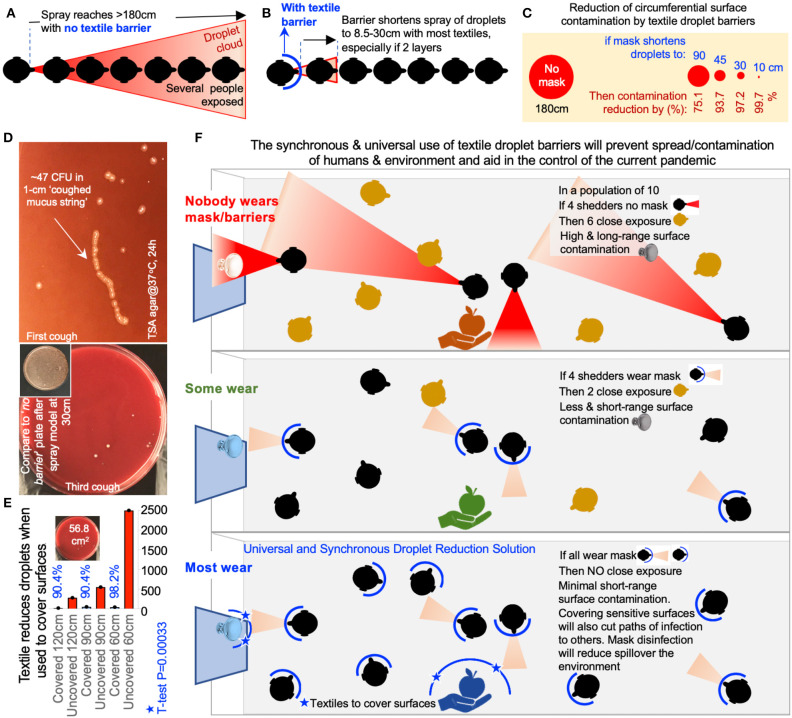
Environmentally-focused “Universal Droplet Reduction Model” against pandemics due to infectious agents transmitted via oral-respiratory fluids. **(A)** Graphical representation of a model where the lack of face barrier/cover could result in the contamination of a large circumferential area, or nearby contact with a higher number of susceptible individuals, within a 180 cm radius. **(B)** Graphical representation illustrating the benefit of wearing textile-face barriers to reduce the circumferential area contaminated with droplets (two-layers/single-layers) and to reduce the number of droplet contacts with susceptible individuals. **(C)** The benefit of using face cover/barriers drastically increases in surface area (cm^2^) as the efficiency of the droplet barrier increases (distance of droplet contamination, cm). **(D)** Coughed material-associated bacteria in agar. Large viscous secretions will be retained by textile-EDB. **(E)** Bacteria-carrying droplet counts on agar plates covered with 1-layer cotton t-shirt material, after one-spray, over distance. Colony-forming units were estimated on paired TSA agar plates (covered and uncovered) following the spraying of the bacterial-carrying solution over the plates, and 48 h of aerobic incubation. **(F)** Environmental droplet reduction model. Protective masks and surface covers in the community. Supplementary Table 2, list of current and proposed actions against COVID-19.

From a surface perspective, if everyone were encouraged to wear EDBs, the collective area contaminated with droplets would be miniaturized to 0.3–2.77% (two-layers/single-layers), compared to the potential contamination within 180 cm (10.2 m^2^). Even suboptimal EDBs, effective for 90 cm radius, could mathematically reduce the EnvDC area by 75.1% (Figures 4A–C). Our findings and surface estimations are conservative as they are based on simulations using a (non-viscous) liquid solution, assuming stationary individuals. However, the impact of EDB is predictably greater since real/large viscous secretions (Figure 4D), which also travel long distances (>180 cm) (23), would be easier to contain by EDB, as communities mobilize. To further lower the risk of fomite (plastic/metal surface) transmission from/by non-EDB-wearers, EDB-textiles used as covers, when relevant, could reduce EnvDC by 90–98% (*T*-test *P* = 0.003, Figure 4E).

Finally, to illustrate in volumetric terms that EDBs are even more effective at preventing EnvDC, we conducted a scoping review of literature to conduct analyses of droplet fluid-carrying capacity. Although published droplet sizes vary with study method (Supplementary Table 1), most sneezed droplets are “large,” and can reach >1 mm. Physiologically, two types of sneeze exist (27): unimodal, when all droplets are large (360 ± 1.5 μm-diameter); and bimodal, when droplets are large (390 ± 1.7 μm-diameter, 70%) and small (72 ± 1.5 μm, 30%). Assuming droplets are spherical, for an average of two sneezes (unimodal:bimodal, 200,000 droplets), we determined that large droplets (85% of total) contain 703-times more fluid than small droplets. Thus, EDBs could reduce COVID-19 EnvDC by effectively blocking the dispersion of fluids/viruses contained in large droplets. Because droplets of <47 μm are known to evaporate before reaching the ground (33), EDB will also prevent small-size droplet aerosolization by trapping such droplets immediately after production. An overview of a “universal textile droplet reduction action-model” against pandemics is illustrated in Figure 4F.

## Discussion

Despite widespread dissemination of information to curtail the rapid spread of COVID-19 outside of China [information which mainly reaches 20–54 year-old adults, who make up 40% of hospitalizations in the USA (34)], little attention has been devoted to EnvDC and prevention strategies for droplet movement from infected to non-infected individuals within the same community. More concerningly is that following mandatory “stay-at-home” quarantine orders, people may return to work unprotected, unaware if they are infected/shedders. This is particularly critical for “essential pandemic workers,” who face different levels of risk (health-care vs. electric/transport/food services), and who can contaminate environmental surfaces as they transit through the community between work (i.e., hospitals) and home, or within their households (35), without wearing masks. Because mass testing is not always possible (6), especially for novel organisms like COVID-19, there are growing concerns that asymptomatic and mildly symptomatic citizens will continue to spread and reintroduce the virus to new areas, creating waves of cases, contributing to further economic burden from the outbreak (36).

Non-pharmaceutical interventions (NPIs), also known as community mitigation strategies, are actions that individuals and communities can take in order to slow the spread of illnesses. For pandemics, when medical approaches (hospitalization/treatments) are limited, NPIs are a critical component to achieve resolution. Although PPE, including masks, are scientifically-effective methods to prevent infectious disease transmission, the use of masks for the general public has not been encouraged by governments (5, 7), possibly because demand will deepen the current crisis of mask unavailability for medical staff, or alternatively, because the use of masks to prevent respiratory infections has been misleadingly deemed ineffective, despite earlier clinical studies indicating that masks could be beneficial in households during pandemics (35, 37, 38).

Although masks have been extensively studied to determine whether individuals are *clinically* protected from infections (39, 40), and to confirm that wearing a mask promotes desirable hygiene practices (handwashing, “avoiding crowds”) (5, 38, 41), masks have not been examined for their potential to *prevent environmental contamination*. Masks work, if worn properly; however, individuals (~50%) often fail to wear masks regularly and properly (37, 42). Despite low compliance, meta-analyses indicate that masks lower the odds of having (SARS)-respiratory infections by 87% (OR = 0.13), compared to the odds of having an infection “not wearing a mask” (43).

Herein, we propose that in addition to seeking the classical/clinical “*prevention of infection,”* NPIs could be universally based on “*droplet reduction models*” such as textile-face covers to mitigate contamination of the environment by respiratory droplets. Not only for the prevention of respiratory diseases, but also to prevent widespread environmental dispersion of the virus, which could reach water sources or affect domestic animals, as has been shown for other viruses, including pandemic influenza (44).

The world was initially in short supply of masks since the international “lockdown” affected production (45), with health-care workers experiencing high morbidity/mortality due to reduced protection (46). Governments have sought private support to increase mask supplies; however, such strategy have taken weeks/months, and infection rates would not improve if supplies were still not available to “lockdown” communities. Increased community transmission leads to higher demand for medical services, unless transmission is halted. Using household textiles is a potentially life-saving cost-effective anti-pandemic strategy because washing/laundering textiles have been shown to destroy COVID-19 by heat (70°C/5 min), bleach (1:49/5 min), and detergents (20 min) (47–50), and is more sustainable (community-level) than using scarce medical disinfectants/supplies. As a rapid solution and alternative to chemical disinfection, and as a step prior to laundering, we highlight the value of ironing (51, 52) because humid and dry heat produced by an iron is safe and in excess of the minimum temperatures needed to destroy viruses and even spore forming bacteria, without affecting the integrity of textile facemasks or face covers. Ironing has been seen as a long-standing disinfection strategy since at least the 1920s (52) and could be universally implemented because most houses have immediate access to, and could safely use, an iron.

Although some materials may allow the passage of more bacteria-containing droplets after three sprays (i.e., compare “Cotton115,” single-layer vs. double, textile with largest mesh pore sizes shown in Supplementary Figure 1), we emphasize that there were no statistical differences attributed to the number of droplets that cross the barrier compared to single-sprays in all the multivariable regression models tested with raw and log_2_-transformed data, especially when tested as two-layers. Collectively, there is no statistical rationale to justify that people should change the mask as a function of number of sneezes to reduce environmental contamination, especially if two-layer masks or covers were used. However, it is advisable to wear/use a clean facemask/surface cover, and that these are cleaned/disinfected (e.g., ironed) after every use, or as often as possible.

To further support the functional value of textiles in public droplet safety, we recently demonstrated *in vivo* that two layers of comb cotton fully protect an environment of germ-free mice and the animals when exposed to up to 20 spray clouds of bacteria-carrying microdroplets (25). Other cotton materials with a less uniform finishing, such as carded yarn, could also provide droplet protection, although this was not tested. The use of homemade coverings combined with household disinfection strategies and information and educational campaigns promoting face cover utilization by the community (e.g., posting door signs) (53) could be more cost-effective compared to the economic effects of prolonged lockdowns. Of note, we emphasize that face covers must be used in conjunction with existing recommendations on hand washing and sneezing into one's arm sleeve.

Sufficient scientific evidence exists (54) and continues to emerge (55–57) to justify the use of face covers to protect the general public not only during the COVID-19 pandemic, but also for every new respiratory virus in the future. While several studies for cloth masks have been conducted with dried aerosols, only a few have studied the impact of wet aerosols. Thus, the present study serves as a reliable, rapid, and reproducible methodology as a platform for liquid droplet testing models. As minor study limitations, we tested only a representative sample of a vast list of potentially available household textiles and did not test dry aerosolized viral particles.

In conclusion, we demonstrated that two-layer household textiles produced a profound reduction of environmental droplet contamination as effectively as medical-grade materials. Encouraging/mandating the synchronous implementation of textile-face covers, while discouraging using medical masks in public, will help control COVID-19.

## Data Availability Statement

The raw data supporting the conclusions of this article will be made available by the authors, without undue reservation, upon request.

## Author Contributions

AR-P envisioned, planned and executed the experiments, analyzed the data, prepared the figures, and wrote the manuscript. SI executed validation and complementary experiments, interpreted the data, and wrote the manuscript. AB assisted with documentation in Supplementary Material and commented and edit the paper. FC and TP commented, revised, and edited the manuscript for medical accuracy and data interpretation. All authors approved the final manuscript.

## Conflict of Interest

The authors declare that the research was conducted in the absence of any commercial or financial relationships that could be construed as a potential conflict of interest.
