# Supplementary material for: Textile Masks and Surface Covers—A Spray Simulation Method and a “Universal Droplet Reduction Model” Against Respiratory Pandemics
Source: Front Med (Lausanne). 2020 May 27;7:260. doi: 10.3389/fmed.2020.00260 (PMC7267001; doi:10.3389/fmed.2020.00260)
Supplement: Supplementary file 1 [file Data_Sheet_1.docx]

**Textile Masks and Surface Covers – A Spray Simulation Method and ‘Universal Droplet Reduction Model’ Against Respiratory Pandemics**

Alex Rodriguez-Palacios, DVM PhD ^1,2^*, Fabio Cominelli, MD PhD ^1,2^,

Abigail R. Basson, RD PhD^1,2^, Theresa T. Pizarro, PhD^3^, and Sanja Ilic, PhD^4^

^1^Division of Gastroenterology & Liver Diseases, Case Western Reserve University School of Medicine and ^2^Digestive Health Research Institute, University Hospitals Cleveland Medical Center, Cleveland, OH, USA; ^3^Department of Pathology, Case Western Reserve University School of Medicine; ^4^Human Nutrition, Department of Human Sciences, College of Education and Human Ecology, The Ohio State University, Columbus, OH, USA.

***Corresponding Author:** Alex Rodriguez-Palacios ([axr503@case.edu](mailto:axr503@case.edu))

**_______________________________________________________________________________**

1. **Supplementary Materials**
2. **Supplementary Figures**
   1. **Supplementary Figure 1.** Textiles and medical materials tested in this study.
   2. **Supplementary Figure 2.** Use of spray-droplet simulation to quantify ability of household textiles to retain droplets.
   3. **Supplementary Figure 3.** Linear regression model to quantify significance of the effect of textiles in spray droplet retention.
   4. **Supplementary Figure 4.** Linear regression coefficients and R^2^ values for CFU-droplet data described in Figure 1d.
   5. **Supplementary Figure 5.** Multinomial regression models to quantify the comparative significance in reduction of droplet count.
   6. **Supplementary Figure 6.** Two 2-layer EDB-textile mask that effectively stops the spread of respiratory droplets in the event of a pandemic could be rapidly fabricated from a piece of clothing.
3. **Supplementary Tables**
   1. **Supplementary Table 1.** Droplet distribution of breath, speech, sneeze and cough
   2. **Supplementary Table 2**. Recommendations to reduce transmission during Covid-19 pandemic
4. **Supplementary References**

**1. SUPPLEMENTARY FIGURES**


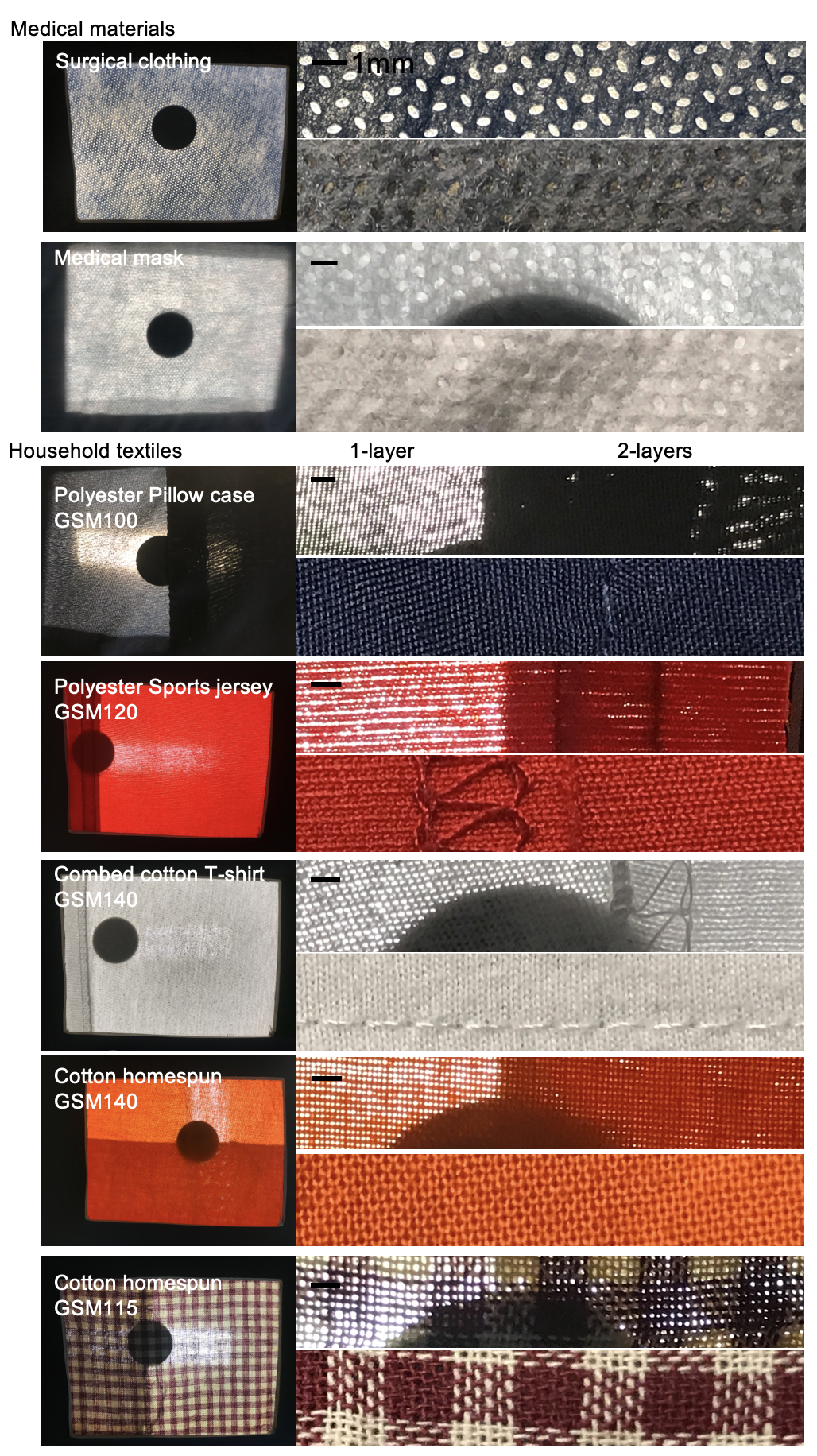


**Supplementary Figure 1. Textiles and medical materials tested in this study.** Bar scale, 1mm. Textiles are shown as a panoramic picture of the textile on the 8.5x11cm window for testing as shown in figure 2A, with transillumination images to visualize the size of the ‘pores’ for the single and double layers. Also, close-up images are shown to illustrate the thread pattern. The number for each textile indicates the grams per share meter.


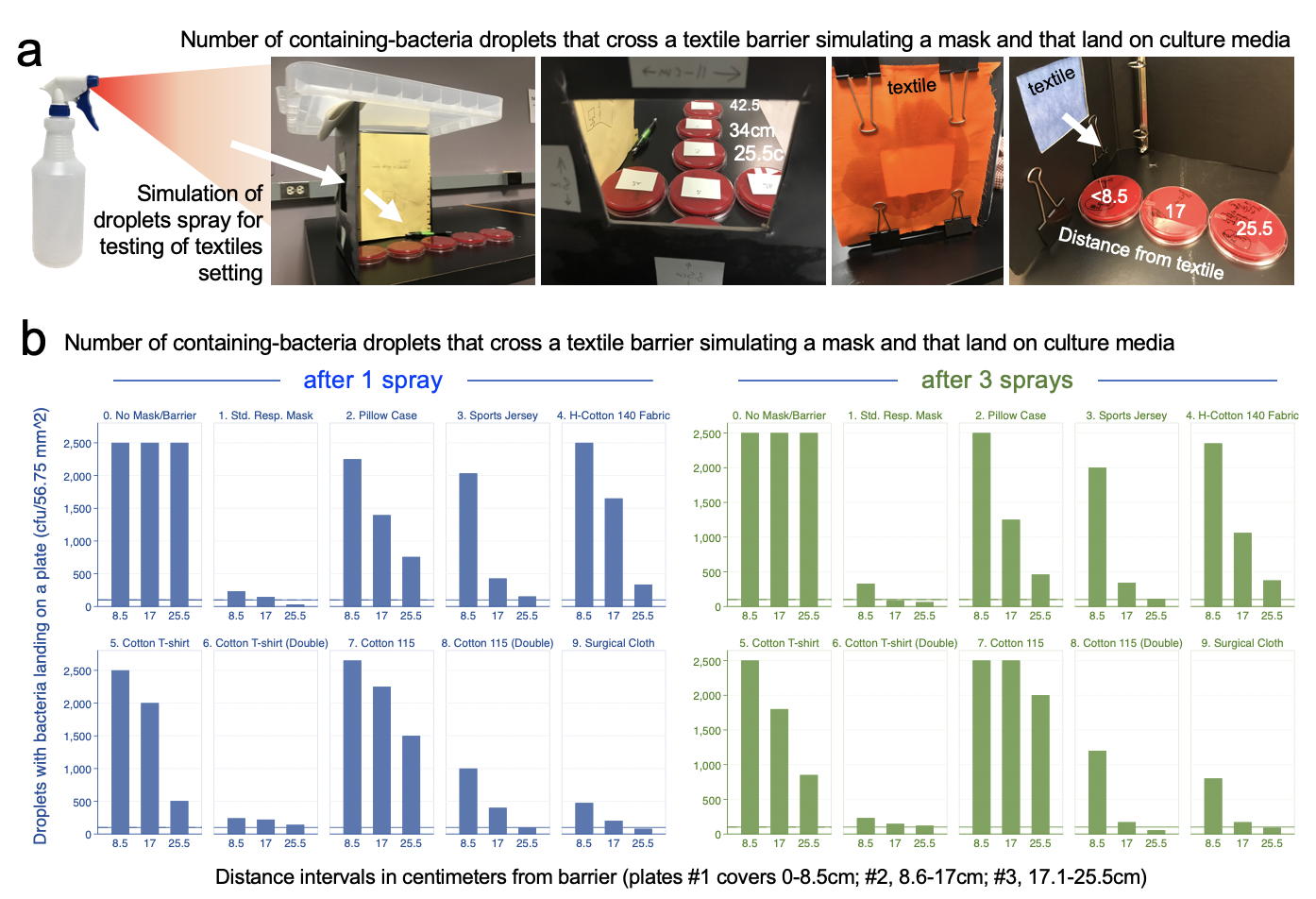


**Supplementary Figure 2. Use of spray-droplet simulation to quantify ability of household textiles to retain droplets. A**) Graphical overview of spray model textile-testing setting also shown in main text. **B**) Bar plots depict mean droplet count illustrating count reduction for various textiles, in single and double layers, at 8.5, 17 and 25.5cm distances from each textile barrier, after 1 and 3 consecutive droplet sprays. Notice increased efficacy using two layers to markedly reduce droplet traffic. Results were reproducible when 1- and 3-spray experiments were tested for each textile (adjusted P>0.48), and when these same experiments were tested at two different institutions (P>0.9 for CFU; P>0.14 for CFU Log_2_ data), despite differences in spray bottles used (**Supplementary Figure 3**). Although some materials may allow the passage of more bacteria-containing droplets after 3 sprays (*i.e.*, compare ‘Cotton115’, single-layer vs. double, textile with largest mesh pore sizes shown in **Supplementary Figure 1**), we emphasize that there were no statistical differences attributed to the number of droplets that cross the barrier compared to single-sprays in all the multivariable regression models tested with raw and log_2_-transformed data, especially when tested as two-layers (see regression models below).


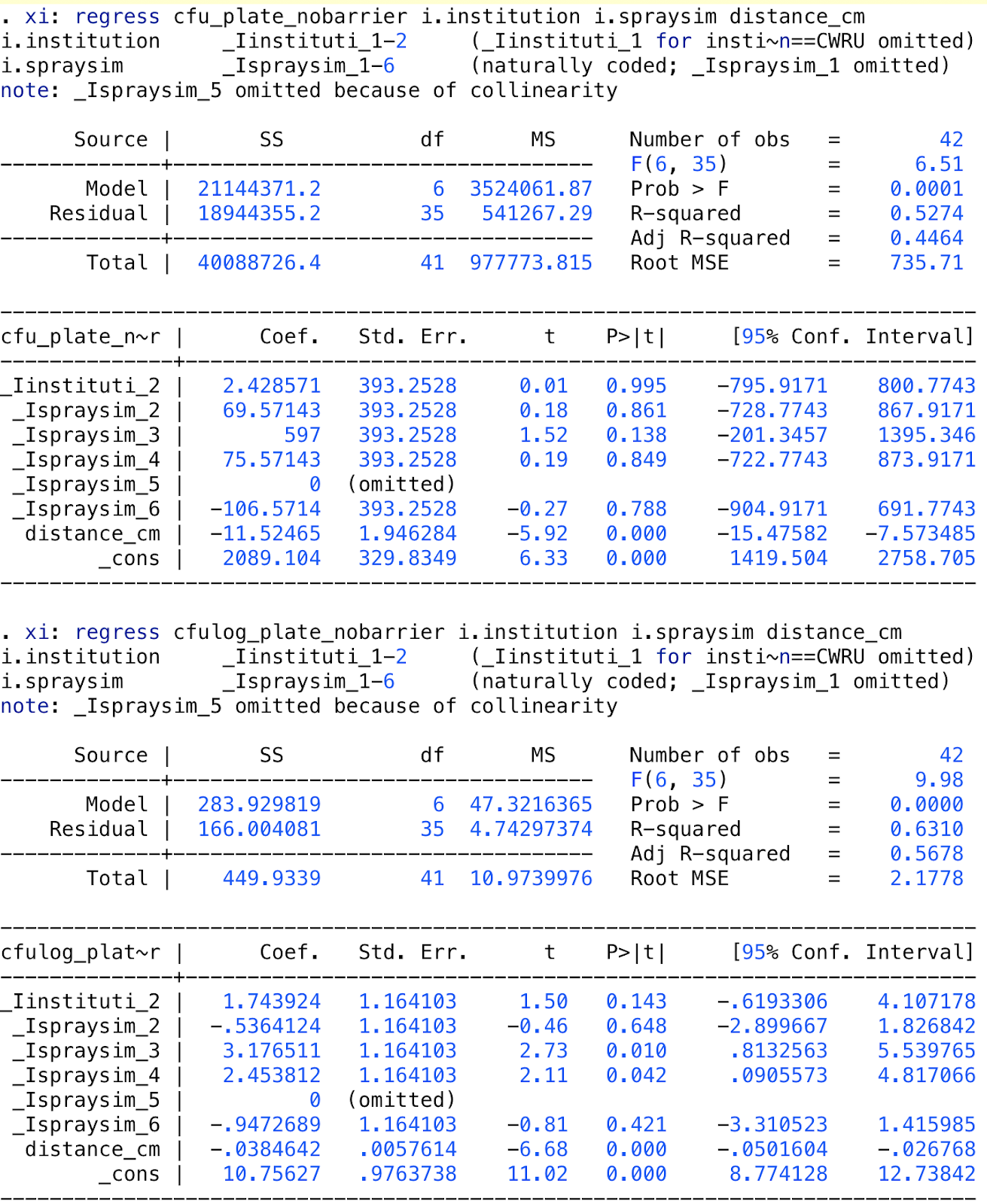


**Supplementary Figure 3.** **Linear regression model to quantify significance of the effect of textiles in spray droplet retention**. Raw data (cfu) and Log2 transformed data (cfulog_plate).


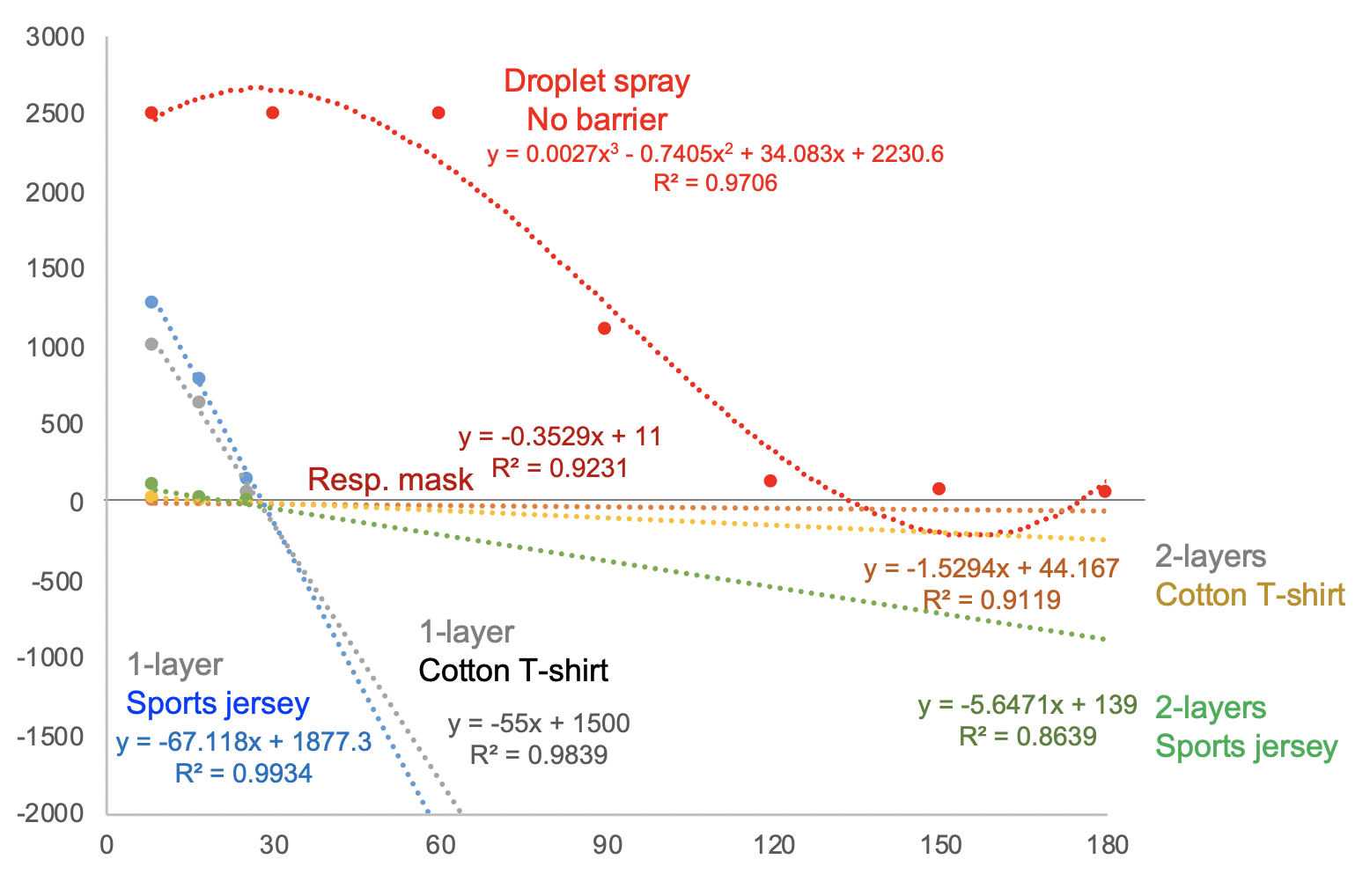


**Supplementary Figure 4.** **Linear regression coefficients and R^2^ values for CFU-droplet data described in** **Figure 2c**. Notice that line slopes closer to the horizontal grid line at 0, and those closer to the ‘Resp. mask’-dotted line, are more effective strategies (2-layers) at inhibiting droplet ejection, compared to single layers.

**
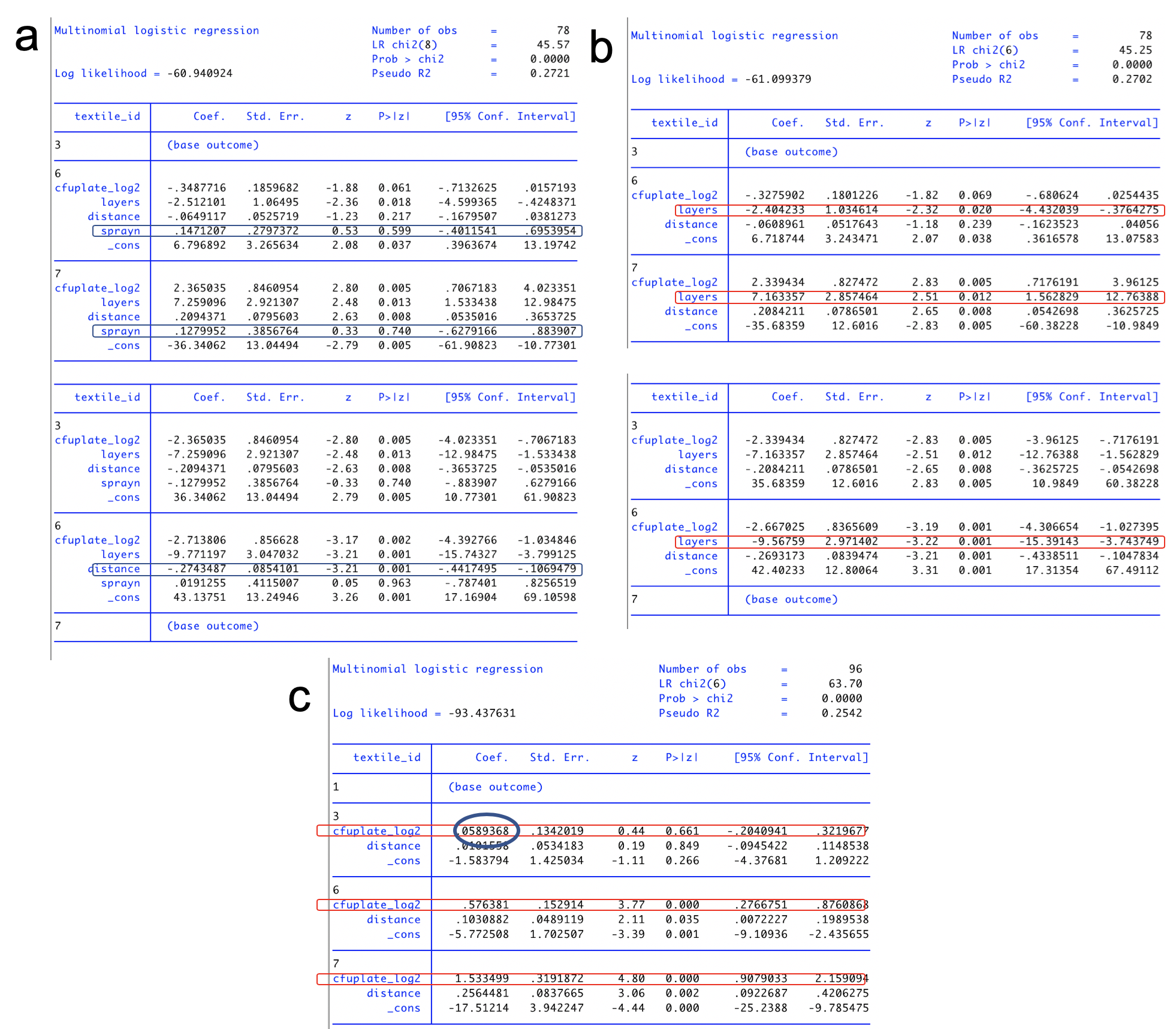
**

**Supplementary Figure 5.** **Multinomial regression models to quantify the comparative significance in reduction of droplet count.** Analysis as a function of number of layers (1 vs. 2), and the number of sprays (1 vs. 3, ‘*sprayn’*) for each of three textiles tested as 2-layers. Log_2_ transformed data (cfulog_plate). **A**) Model including *sprayn*. Notice p-values indicate no difference between 1 and 3 sprays regarding ability of textiles to retain droplets. This indicates that a very humid textile will not result in more droplets, if sprayed. **B**) Model to interpret effect of *layers*, after removal of *sprayn*, since there was no effect on droplet counts. Coefficient indicates the number of Log_2_ unit difference for each model compared to comparator base. **C**) Model to visualize similarity of 2-layer textiles to respiratory mask. All analyses were conducted in a blind fashion (codes released when analysis completed). Textile#1, respiratory mask; #3, sports jersey; #6, cotton T-shirt; #7, cotton homespun 115.


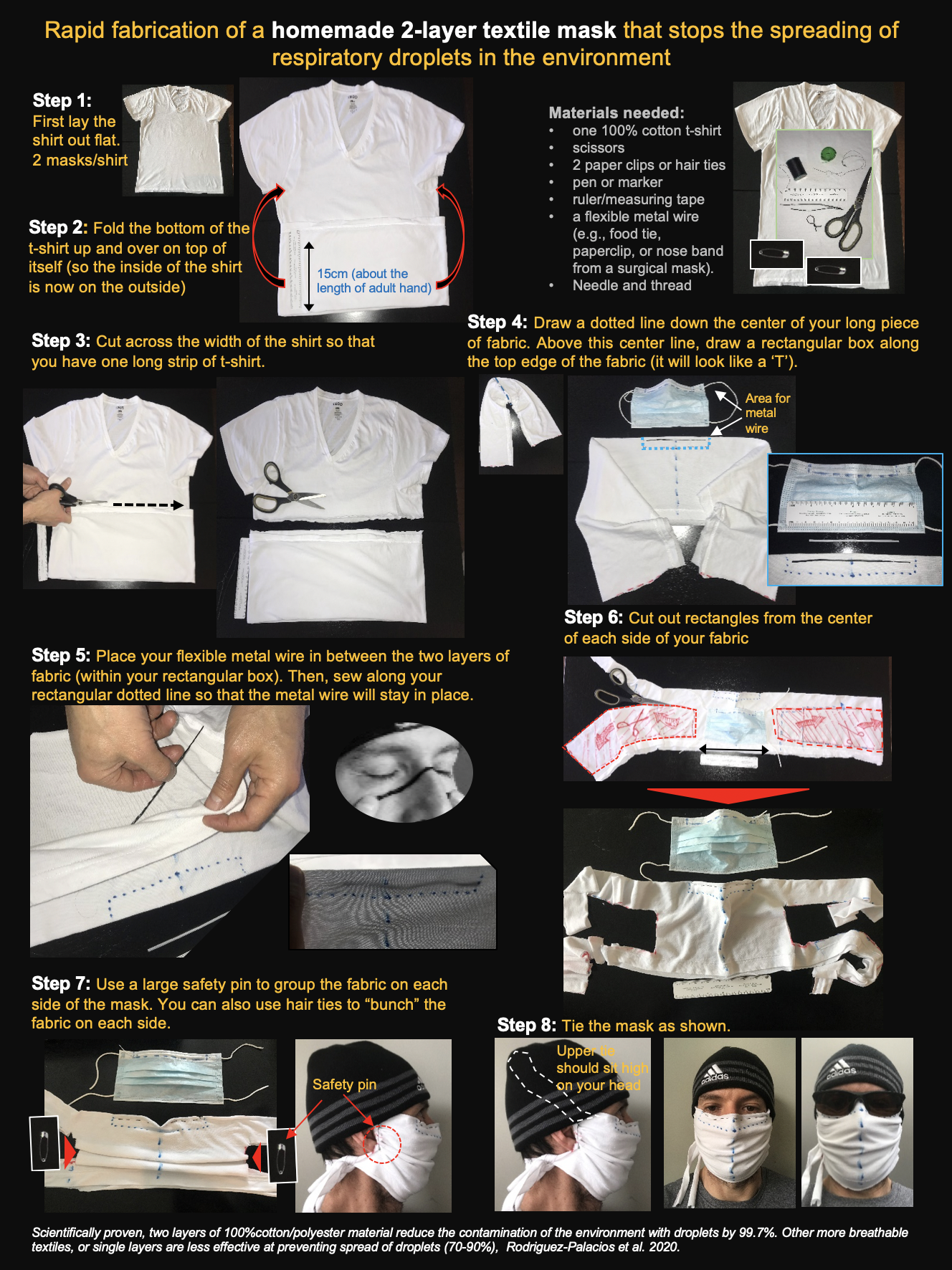


**Supplementary Figure 6.** **Two 2-layer EDB-textile mask that effectively stops the spread of respiratory droplets in the event of a pandemic could be rapidly fabricated from a piece of clothing.** When masks are used, it is important that they are used properly and guidelines for appropriate handling of masks are available[1, 2]. Even if not fully efficient, wearing masks has been shown to promote other preventive hygiene practices that control infections, *e.g.,* handwashing, and ‘avoiding crowds’[3]. Masks also serve as visual reminders for others not to touch their nose/eyes and then spread virus to surfaces, restricting the paths of disease transmission.

**3. SUPPLEMENTARY TABLES**

**Supplementary Table 1.** Droplet distribution of breath, speech, sneeze and cough

|  | **Droplet size, um** | **Number of droplets** |  | **Total mass, mg (mask)** | **Total mass, mg (plastic)** |
| --- | --- | --- | --- | --- | --- |
| **Breath** | <1 | 12.5/L for <1um and 1.9/L for >1um[4] |  |  |  |
| **Speech** | 3-1500 | ~7 per word (3.4±2.7particles/second) |  | 18.7mg | 79.4mg |
| **Sneeze** | 1-2000 | ~40,000 |  |  |  |
| **Cough** | 1-2000 | ~800[5] |  | 22.9mg | 85 mg |
| Table adapted from Han *et al*.[6] and data from Xie *et al*[5]. Literature has variable ranges because of the method of technology used to measure. | | | | | |

**Supplementary Table 2**. Environmental Droplet Reduction Barriers (EDBs) and expanded new proposed recommendations to reduce the contamination of the environment with respiratory droplets and thus reduce the risk of transmission/acquisition of Covid-19 during pandemic status

| **WHO/CDC current recommendations (community-level)**[7-10] |
| --- |
| Frequent hand hygiene (alcohol-based rub, washing soap & water). |
| Avoid touching your eyes, nose, mouth.* |
| Practice good respiratory behaviors (e.g., sneeze into elbow, tissue disposal) |
| If sick (symptomatic individual), wear a medical mask. |
| Wash hands after disposing of a used medical mask. |
| Maintain social distancing (minimum 6 feet; 2 meters) from persons with symptoms. |
| Clean frequently touched surfaces and objects daily (e.g., tables, light switches, doorknobs). |
| **WHO/CDC current public health measures**[7-10] |
| Isolation and treatment of ill individuals. |
| Monitoring symptoms of healthy contacts. |
| Traveler health advice. |
| Environmental cleaning. |
| Avoid crowding (mass gatherings). |
| Consider school, transport, workplace closures. |
| Consider public health quarantine. |
| **Proposed new recommendations (personal)** |
| Wearing a homemade textile EDB facemask barriers for *ALL* persons. |
| Bend slightly forward when sneezing. Sneeze/cough into angle of flexed elbow. |
| *Face hygiene**: wash face when returning home. |
| *Hair hygiene*: cover hair with hat. |
| *Eye hygiene*: cover eyes with glasses/sunglasses/goggles. |
| *Clothing hygiene*: change & wash clothes when returning home. |
| *Food hygiene*: when eating, cover mouth with napkin if talking. Talking eating/outloud release droplets in the environment.  Cover surfaces with textiles and practice frequent cleaning since droplets last longer on plastic and metallic surfaces. |
| **Proposed new recommendations (environmental)** |
| Avoid touching public surfaces (e.g., handrails). |
| Use a secondary object (e.g., key, pen) to press elevator buttons. |
| Clean frequently touched surfaces, in particular plastics and metal. |
| *Surface hygiene 1:* cover surfaces with textiles & wash frequently. |
| *Surface hygiene 3:* disinfect** groceries and deliveries. |
| **Proposed new recommendations (institutional)** |
| Create training programs of members of institutions on ‘respiratory droplets and environmental droplet safety.  Reinforce education by posting 'public droplet precaution' signs in public areas (e.g., elevator). |
| Educate and promote the constant use of homemade textile EDB-facemask barriers, over purchasing medical masks, to reduce environmental contamination for all, even healthy, individuals, while the local/global pandemic status/measures deem it necessary. |
| Table developed from WHO [7-9] and CDC[10] guidance documents.  *On average, a person touches their face 23 times/hour.[11, 12]  **Disinfecting can be performed using the following: ethanol-based spray, diluted bleach, most household cleaning products, and/or washing with soap and water for at least 20 seconds.  See **Supplementary Discussion** on the use of masks**.**  Our recent studies support the effective prevention potential of homemade masks rapidly fabricated using widely available cotton fabrics[13]. The U.S. Centers for Disease Control now provides guidance for sewn and non-sewn versions, and tutorials[14]. |

**Supplementary References**

1. Liu, Z., et al., Understanding the factors involved in determining the bioburdens of surgical masks. Ann Transl Med, 2019. **7**(23): p. 754.

2. WHO., *World Health Organization. Coronavirus disease (COVID-19) advice for the public: When and how to use masks.* Available at: <https://www.who.int/emergencies/diseases/novel-coronavirus-2019/advice-for-public/when-and-how-to-use-masks>. Accessed March 18, 2020. 2020.

3. Wada, K., K. Oka-Ezoe, and D.R. Smith, Wearing face masks in public during the influenza season may reflect other positive hygiene practices in Japan. BMC Public Health, 2012. **12**: p. 1065.

4. Papineni, R.S. and F.S. Rosenthal, The size distribution of droplets in the exhaled breath of healthy human subjects. J Aerosol Med, 1997. **10**(2): p. 105-16.

5. Xie, X., et al., Exhaled droplets due to talking and coughing. J R Soc Interface, 2009. **6 Suppl 6**: p. S703-14.

6. Han, Z.Y., W.G. Weng, and Q.Y. Huang, Characterizations of particle size distribution of the droplets exhaled by sneeze. J R Soc Interface, 2013. **10**(88): p. 20130560.

7. World Health Organization, *Rational use of personal protective equipment (PPE) for coronavirus disease (COVID-19). Interim guidance; March 19, 2020.* Available at: <https://www.who.int/emergencies/diseases/novel-coronavirus-2019/technical-guidance/critical-preparedness-readiness-and-response-actions-for-covid-19>.

8. World Health Organization, *Responding to community spread of COVID-19. Interim guidance;  March 7, 2020.* Available at: <https://www.who.int/emergencies/diseases/novel-coronavirus-2019/technical-guidance/critical-preparedness-readiness-and-response-actions-for-covid-19>.

9. World Health Organization, *Operational considerations for case management of COVID-19 in health facility and community. Interim guidance;  March 7, 2020. Available at:* <https://www.who.int/emergencies/diseases/novel-coronavirus-2019/technical-guidance/critical-preparedness-readiness-and-response-actions-for-covid-19>.

10. Centers for Disease Control and Prevention. *Coronavirus Disease 2019 (COVID-19). Get Your Home Ready. Accessed March 18, 2020.* <https://www.cdc.gov/coronavirus/2019-ncov/prepare/get-your-household-ready-for-COVID-19.html>.

11. Kwok, Y.L., J. Gralton, and M.L. McLaws, Face touching: a frequent habit that has implications for hand hygiene. Am J Infect Control, 2015. **43**(2): p. 112-4.

12. Shannon, J., *USA today. Health officials say not to touch your face. That's harder than it sounds – even for them. Accessed March 26, 2020.* <https://www.usatoday.com/story/news/health/2020/03/08/coronavirus-prevention-how-stop-touching-your-face/4965517002/>.

13. CDC, *Use of Cloth Face Coverings to Help Slow the Spread of COVID-19. Coronavirus Disease 2019 (COVID-19). April 4, 2020. Uso de cubiertas de tela para la cara para ayudar a desacelerar la propagación del COVID-19.* Available at <https://www.cdc.gov/coronavirus/2019-ncov/prevent-getting-sick/diy-cloth-face-coverings.html>*. .* 2020.

14. Adams, J., *Surgeon General Shows How to Make Your Own Face Covering. Surgeon General  Dr. Jerome Adams. Tutorial April 4, 2020.* Available at <https://www.youtube.com/watch?v=PI1GxNjAjlw>. 2020.
